# Mechanisms of neutralization of toxSAS from toxin–antitoxin modules

**DOI:** 10.1038/s41589-024-01630-4

**Published:** 2024-06-04

**Authors:** Lucia Dominguez-Molina, Tatsuaki Kurata, Albinas Cepauskas, Dannele Echemendia-Blanco, Safia Zedek, Ariel Talavera-Perez, Gemma C. Atkinson, Vasili Hauryliuk, Abel Garcia-Pino

**Affiliations:** 1 Cellular and Molecular Microbiology, Faculté des Sciences, Université libre de Bruxelles (ULB), Brussels, Belgium; 2 Department of Experimental Medical Science, Lund University, Lund, Sweden; 3 Faculty of Science and Technology, University of Tartu Institute of Technology, Tartu, Estonia; 4 Science for Life Laboratory, Lund, Sweden

**Keywords:** Translation, X-ray crystallography, Bacteria, Enzyme mechanisms

## Abstract

Toxic small alarmone synthetase (toxSAS) enzymes constitute a family of bacterial effectors present in toxin–antitoxin and secretion systems. toxSASs act through either translation inhibition mediated by pyrophosphorylation of transfer RNA (tRNA) CCA ends or synthesis of the toxic alarmone adenosine pentaphosphate ((pp)pApp) and adenosine triphosphate (ATP) depletion, exemplified by FaRel2 and FaRel, respectively. However, structural bases of toxSAS neutralization are missing. Here we show that the pseudo-Zn^2+^ finger domain (pZFD) of the ATfaRel2 antitoxin precludes access of ATP to the pyrophosphate donor site of the FaRel2 toxin, without affecting recruitment of the tRNA pyrophosphate acceptor. By contrast, (pp)pApp-producing toxSASs are inhibited by Tis1 antitoxin domains though occlusion of the pyrophosphate acceptor-binding site. Consequently, the auxiliary pZFD of AT2faRel is dispensable for FaRel neutralization. Collectively, our study establishes the general principles of toxSAS inhibition by structured antitoxin domains, with the control strategy directly coupled to toxSAS substrate specificity.

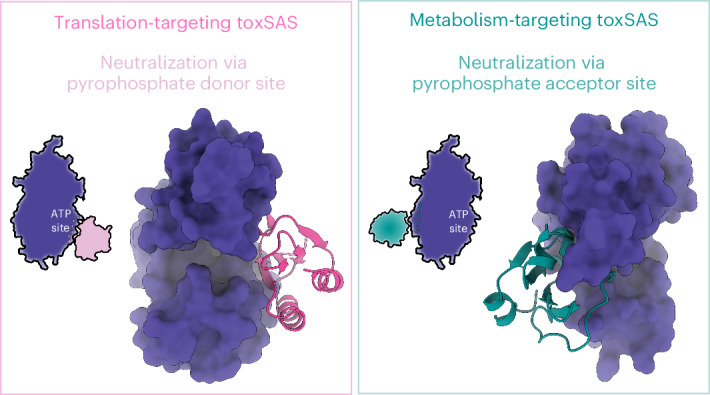

## Abstract (web summary)

Toxic small alarmone synthetase (toxSAS) enzymes are toxic effectors of certain toxin–antitoxin modules, involved in phage defense and secretion systems. Here the authors establish the mechanism underlying toxSAS inhibition by structured antitoxins and reveal the connection between neutralization strategy and substrate specificity.

## Main

Small alarmone synthetases (SASs) are a diverse group of monofunctional RelA/SpoT Homolog (RSH) enzymes that catalyze the transfer of the adenosine triphosphate (ATP)-derived pyrophosphate moiety on the 3′ ribose position of the acceptor substrate^1^. The acceptor substrate is commonly a purine nucleotide, either guanosine triphosphate, diphosphate or monophosphate (GTP, GDP or GMP, yielding guanosine pentaphosphate ((pp)pGpp) alarmone nucleotides)^2–4^ or their adenosine equivalents (ATP, ADP or AMP, yielding (pp)pApp)^5,6^. SASs also catalyze pyrophosphate transfer to the adenine moiety of the 3′ CCA end of transfer RNAs (tRNAs), yielding pyrophosphorylated tRNA (tRNA-PP)^7^. As such, the substrate specificity of SASs is broadly determined by their biological function; guanosine-specific SASs are bacterial stress response factors that modulate the intracellular levels of the (pp)pGpp alarmone^3,8,9^, while adenine-specific toxic SASs (toxSASs) are potent inhibitors of bacterial growth^5,7,10,11^. The accumulation of toxSAS-produced (pp)pApp abrogates ATP production^5,6^, while pyrophosphorylation of tRNA CCA abrogates protein synthesis by rendering the modified tRNA aminoacylation incompetent^7^.

Due to their extreme toxicity, the enzymatic activity of toxSASs is tightly controlled. Acting in trans, dedicated immunity proteins and antitoxins sequester toxSASs into inactive complexes^5–7,11^. Acting in cis, the pseudo-Zn^2+^ finger domain (pZFD) autoinhibitory antitoxin element of the monomeric fused single-polypeptide toxin–antitoxin system (TA) CapRel^SJ46^ inhibits its toxic enzymatic domain (toxSYNTH)^10^. The pZFD^CapRel^ was predicted to block access of ATP, the pyrophosphate donor, through a conserved YXXY sequence motif that switches to a 3_10_-helical conformation when anchored to the toxSYNTH of CapRel^SJ46^ for neutralization^10^. Furthermore, pZFD^CapRel^ also acts a phage sensor domain that mediates the activation of the toxin via the recognition of the phage SECΦ27 major capsid protein Gp57^10^. The proposed disengagement of pZFD^CapRel^ from the toxSYNTH active site enables CapRel^SJ46^-mediated tRNA pyrophosphorylation, ultimately resulting in translational shutoff, which quarantines the infected cell^10^.

A different regulatory strategy is used to control *Pseudomonas aeruginosa* PAO1 Tas1 (type VI secretion effector (p)ppApp synthetase 1), which contains a divergent toxSAS-related domain^6^. Unlike the tRNA-targeting FaRel2 and CapRel^SJ46^, Tas1 produces the toxic alarmone (pp)pApp, a potent inhibitor of purine biosynthesis, which depletes the cellular ATP pool^6^. Tas1 is neutralized by the immunity factor Tis1 (type VI secretion immunity to (p)ppApp synthetase 1). The formation of the Tas1:Tis1 heterodimer complex is proposed to block access of the pyrophosphate acceptor, not donor, ATP nucleotide to the toxSYNTH domain of Tas1 (ref. ^6^). The toxic TA effector *Cellulomonas marina* FaRel is a well-characterized (p)ppApp synthetase^5^. The *faRel* gene is encoded in an operon with a structure that is highly uncommon for typically bicistronic TAs; it is flanked by two antitoxin genes: *aTfaRel*, encoding a small alarmone hydrolase (SAH) enzyme that can promiscuously neutralize the toxic products of toxSASs, and *aT2faRel*, encoding a type II TA-like antitoxin specific to FaRel^5^.

The *Coprobacillus* sp. D7 *faRel2*–*aTfaRel2* operon encodes a type II TA system, with the FaRel2 toxSAS toxin being specifically neutralized by the ATfaRel2 antitoxin^5^ (Supplementary Fig. 1a–c). FaRel2 is a protein synthesis inhibitor that pyrophosphorylates uncharged tRNAs, similarly to CapRel^SJ46^ (ref. ^7^). As the ATfaRel2 antitoxin is a distant homolog of pZFD^CapRel^, it is also likely to prevent the binding of ATP to the pyrophosphate donor site of the toxSYNTH domain^10^. However, direct structural evidence for the mode of neutralization of either fused (CapRel^SJ46^) or bipartite (FaRel2:ATfaRel2) translation-targeting toxSAS TAs is lacking^5,7^.

Here we uncover how tRNA-modifying toxSAS are neutralized. We determine the crystal structure of the individual FaRel2 toxin and ATfaRel2 antitoxin, as well as the heterotetrameric FaRel2_2_:ATfaRel2_2_ complex. Our structural, microbiological and biochemical evidence suggests that bipartite toxSAS TA operons use stable multimerization as an alternative to the colocalization of toxin and antitoxin domains of monomeric fused TAs, such as CapRel^SJ46^, to ensure efficient toxin inhibition. We directly demonstrate that ATfaRel2 neutralizes FaRel2 by blocking access of ATP to the pyrophosphate donor site of toxSYNTH. Lastly, we propose a unifying conceptual framework that connects the mechanisms of allosteric control and substrate specificity in toxSASs.

## Results

### ATfaRel2 is a compact, well-structured antitoxin

**Fig. 1:**
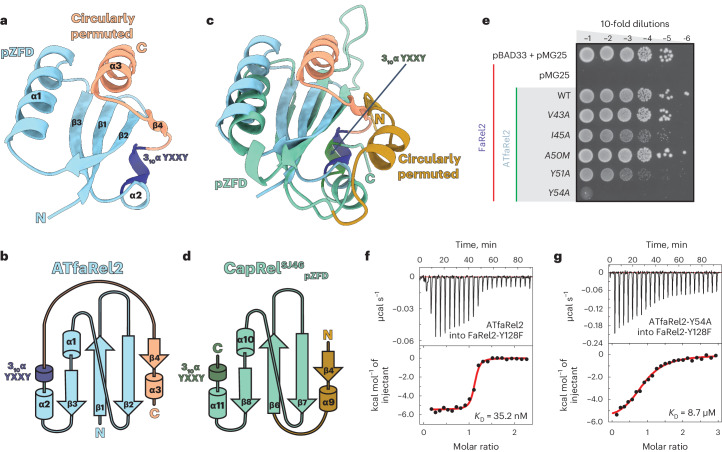
Structure of the ATfaRel2 antitoxin. **a**, Cartoon representation of *Coprobacillus* sp. D7 ATfaRel2 structure. The pZFD core is colored light blue, the C-terminal extension elements are colored salmon and the ^51^YXXY^54^ motif is highlighted in dark blue. **b**, Topology representation of ATfaRel2, colored as in **a**. **c**, Superposition of the experimental ATfaRel2 structure (colored as in **a**) onto the AlphaFold2-predicted structure of the CapRel^SJ46^ antitoxin domain in the neutralizing state. pZFD^CapRel^ is colored light green and the N-terminal extension that is part of Anchor1 is colored ocher, with the conformational switch region that contains the YXXY neutralization motif colored dark green and labeled in the figure. **d**, Topology representation of the CapRel^SJ46^ antitoxin in the neutralizing state, highlighting the circular permutation of ATfaRel2. **e**, Toxicity neutralization assays probing the ^51^YXXY^54^ motif of ATfaRel2 and its scaffolding structure from β1. Serial dilutions of *E.* *coli* strains expressing WT FaRel2 alone or coexpressed with ATfaRel2 (WT or V43A, I45A, A50M, Y51A and Y54A variants) were plated on solid LB medium and scored after 16 h at 37 °C. **f**,**g**, Binding of WT (**f**) and Y54A-substituted (**g**) ATfaRel2 to Y128F-substituted catalytically compromised FaRel2, monitored by ITC.

We determined the crystal structure of ATfaRel2 (residues 1–73) to 1.2-Å resolution (Fig. 1a,b and Supplementary Table 1). The antitoxin protein is monomeric in the crystal (Supplementary Fig. 2a), in good agreement with molecular weight estimates by size-exclusion chromatography (SEC) (Supplementary Fig. 2b and Supplementary Table 2). At the core of its compact, well-folded structure is an antiparallel β-sheet (β-strands β1–β3), with β2 and β3 connected by the central α-helix α1. The C-terminal extension provides an additional β-strand to the β-sheet, β4, which folds parallel to β2, as well as a second α-helix, α2, which forms a part of the protein’s hydrophobic core (Fig. 1b). Despite the lack of sequence similarity, ATfaRel2 is structurally similar to pZFD^CapRel^, with the two proteins superimposing with a root-mean-square deviation (r.m.s.d.) of 2.8 Å, and they both contain conserved tyrosine residues at the C terminus (Fig. 1c).

Interestingly, the predicted topologies of pZFD^CapRel^ in the neutralizing state and ATfaRel2 are similar except for the order of structural elements at the termini. The C-terminal β/α extension of ATfaRel2 is structurally similar to the N-terminal β/α region of pZFD^CapRel^ that connects the antitoxin with toxSYNTH^CapRel^ via Anchor1 (Fig. 1c and Supplementary Fig. 2c–e). This is suggestive of circular permutation (compare Fig. 1b to Fig. 1d). However, as their similarities extend only to the topology level, these two small β/α elements may have evolved independently.

Changes in topology in the antitoxin elements likely reflect the in cis versus in trans neutralization of different toxSASs. Presumably, ATfaRel2 fully dissociates from FaRel2 when the toxin becomes active. Conversely, in the fused CapRels, dissociation of pZFD^CapRel^ is not possible and activation is believed to be mediated by a conformational change^10^ (Supplementary Fig. 2d,e). Despite these topological variations, both domains retain strong structural similarities, including the YXXY sequence motif linked to toxSYNTH inhibition in CapRel^SJ46^. This suggests that ATfaRel2 and pZFD^CapRel^ neutralize toxSYNTH domains by a common mechanism.

### The YXXY motif is crucial for toxSYNTH inhibition

In CapRel^SJ46^, the conserved YXXY motif is located in the switch region of the pZFD and is predicted to lock the enzyme in a catalytically inactive state with both Y residues blocking the donor pyrophosphate ATP-binding site of toxSYNTH (Supplementary Fig. 2c–e). Substitutions directly or indirectly targeting this motif render CapRel^SJ46^ constitutively active^10^. In the crystal structure of the catalytically active state of CapRel^SJ46^, the YXXY motif assumes an extended conformation^10^. Conversely, in the AlphaFold2-generated neutralized state model, the YXXY motif is predicted to fold into a 3_10_-helix that locks into the donor site (Supplementary Fig. 2d,e).

The structure of the free ATfaRel2 reveals that the ^51^YXXY^54^ motif indeed folds into a 3_10_-helix structure that was predicted to be key for toxSAS neutralization, scaffolded by β-strands β1 and β3 (Fig. 1a and Supplementary Fig. 1b). This suggests that, even in the absence of the toxin, ATfaRel2 is primed for efficient FaRel2 neutralization. Guided by the structural similarities between ATfaRel2 and pZFD^CapRel^, we probed the role of the individual residues of the ^51^YXXY^54^ motif in the neutralization of FaRel2 toxicity in vivo (Fig. 1e). While Y54A substitution fully ablates the neutralizing activity of ATfaRel2, I45A and Y51A result in modest but clearly detectable defects in FaRel2 neutralization. Our isothermal titration calorimetry (ITC) assays lend further support to the in vivo neutralization results. To overcome the challenges of producing an otherwise highly toxic FaRel2, we followed a well-established substitution strategy for RSH enzymes^10,12,13^ and we used a catalytically impaired Y128F-substituted variant, FaRel2-Y128F. The substitution interferes with the accommodation of the acceptor nucleotide in the active site but is located far from the predicted pZFD^CapRel^–toxSYNTH interface. We characterized complex formation between FaRel2-Y128F and wild-type (WT) ATfaRel2 or variants carrying substitutions in the ^51^YXXY^54^ motif (Fig. 1f,g and Supplementary Table 3). V43A and A50M substitutions had a negligible effect on complex stability. These mutant antitoxins bound FaRel2-Y128F with *K*_D_ values of 78.8 nM and 38.0 nM, respectively, compared with a *K*_D_ value of 35.2 nM in the case of WT ATfaRel2. The destabilizing effect was more pronounced in the case of I45A and Y51A variants. While these substitutions resulted in 25-fold and 43-fold reductions in affinity, respectively, the remaining affinity was still sufficient for partial neutralization in vivo (Fig. 1e). Lastly, the Y54A variant, which was unable to neutralize FaRel2 in vivo, had a 250-fold lower affinity to the toxin than WT ATfaRel2.

### FaRel2 binds ATP in a partially folded state

**Fig. 2:**
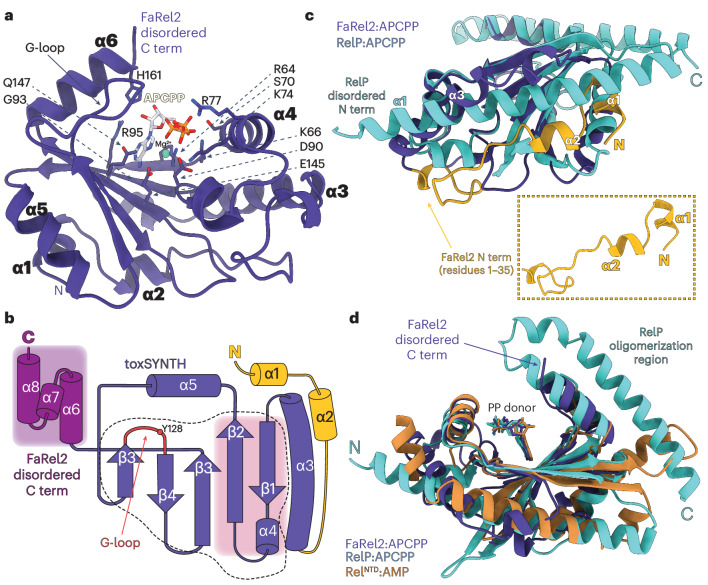
Structure of FaRel2 in complex with APCPP. **a**, The structure of *Coprobacillus* sp. D7 FaRel2 in complex with APCPP bound to the pyrophosphate donor site. The adenine base is coordinated by conserved residues R64, R95 and Q147, as well as residue G93, while E145 provides the specificity for adenosine nucleotides. R64 provides additional van der Waals contacts with the ribose moiety. The 5′ triphosphate moiety is stabilized by R64, K66, S70, K74 and R77, while the Mg^2+^ ion is held in place by the catalytic D90. **b**, Topology diagram of FaRel2, highlighting the N-terminal region involved in tRNA recognition (yellow), the toxSYNTH domain (dark blue, with the catalytic region outlined by a dashed line) and the disordered C-terminal region (dark magenta). The acceptor site is highlighted by the G-loop (β3–β4 loop) in red and the donor site involving β1, β2 and α3 is shaded in pink. **c**, Superposition of the FaRel2:APCPP complex on the structure of *S.* *aureus* RelP in complex with APCPP (PDB 6EWZ; turquoise). The ordered N terminus of FaRel2 that is lacking in RelP is highlighted in yellow. **d**, Superposition of the complexes of FaRel2:APCPP (dark blue), RelP:APCPP (turquoise) and *Thermus thermophilus* Rel^NTD^:AMP (dark orange; PDB 6S2U) illustrates the conserved mode of pyrophosphage donor coordination in RSH pyrophosphotransferases.

Next, we determined the structure of the catalytically impaired FaRel2-Y128F bound to the non-hydrolyzable ATP analog adenosine-5′-[(α,β)-methyleno]triphosphate (APCPP) at 2.6-Å resolution (Fig. 2a). The toxSYNTH domain of FaRel2 shares the overall topology of other nucleotide pyrophophotransferases, retaining a presumably ancestral fold composed of a central five-stranded β-sheet framed by two α-helices, α3 and α6 (refs. ^13,14^) (Fig. 2b). The core toxSYNTH domain is very similar to that of the (pp)pApp alarmone synthetase Tas1 (Protein Data Bank (PDB) 6OX6) and the tRNA-pyrophosphokinase CapRel^SJ46^ (PDB 7ZTB), superimposing with r.m.s.d. values of 1.0 Å and 0.8 Å, respectively (Supplementary Fig. 3a,b).

The catalytic core of RSH enzymes is typically decorated by regulatory elements that exert allosteric control on the enzymatic domains^12,15^. The FaRel2 toxSYNTH domain has a well-resolved N-terminal α-helical extension comprising helices α1 and α2, with residues K28 and R29 from the α2–α3 loop being crucial for tRNA binding^7^ (Supplementary Fig. 1c). While the N terminus is unresolved (Fig. 2c) in the non-toxic (p)ppGpp alarmone synthetases RelP^15,16^ and RelQ^17^, the N terminus folds back toward the toxSYNTH core in FaRel2 and intercalates α1 and α2 between α3 and α5, providing an anchor point for tRNAs near the active site G-loop (Fig. 2a,c). The C-terminal region of SYNTH and toxSYNTH domains of pyrophosphotransferases have varied architectures composed predominantly of small α-helical domains^6,10,13,14,18,19^; the C-terminal bundle of four α-helices of SASs RelQ and RelP, which synthesizes (p)ppGpp, acts as an oligomerization interface^15,17^, toxSYNTH of the monomeric Tas1 is followed by a small α-helical domain^6^ and the SYNTH domain of long RHSs is followed by a flexible core domain with a high α-helical propensity^18^. In the case of FaRel2 complexed with APCPP, the C terminus is disordered and not visible in the electron density (Fig. 2d).

Despite these differences, the FaRel2-bound APCPP superimposes remarkably well with the APCPP bound in the donor site of other (p)ppGpp-synthesizing RSHs SAS RelP and RelQ, as well as the long RSH Rel^13,15,17^ (Fig. 2d). As in the other alarmone synthetases, the adenosine group stacks with the conserved R64 and R95 from β1 and β2, with β5 E145 hydrogen bonding the adenosine NH_2_ group (Supplementary Fig. 1c). The R64 residue has a crucial role in providing van der Waals contacts to accommodate the ribose while directly coordinating the 5′ α and β phosphates together with K66. The strongly basic α4 that follows β1 further stabilizes the triphosphates through S70, K74 and R77 (Fig. 2a). At the pyrophosphate acceptor side of the active site, the conformation of the G-loop and the orientation of the base-coordinating F128 (Y128 in the WT FaRel2) deviate from what was observed in precatalytic and postcatalytic complexes of (p)ppGpp synthetases^15,17^ (Supplementary Fig. 3c,d). It is tempting to speculate that, while the ground state of FaRel2 is primed to bind ATP, efficient tRNA binding would likely involve further conformational rearrangements, such as folding of the C terminus and alignment of the active site.

### ATfaRel2 binds FaRel2 by β-sheet extension

**Fig. 3:**
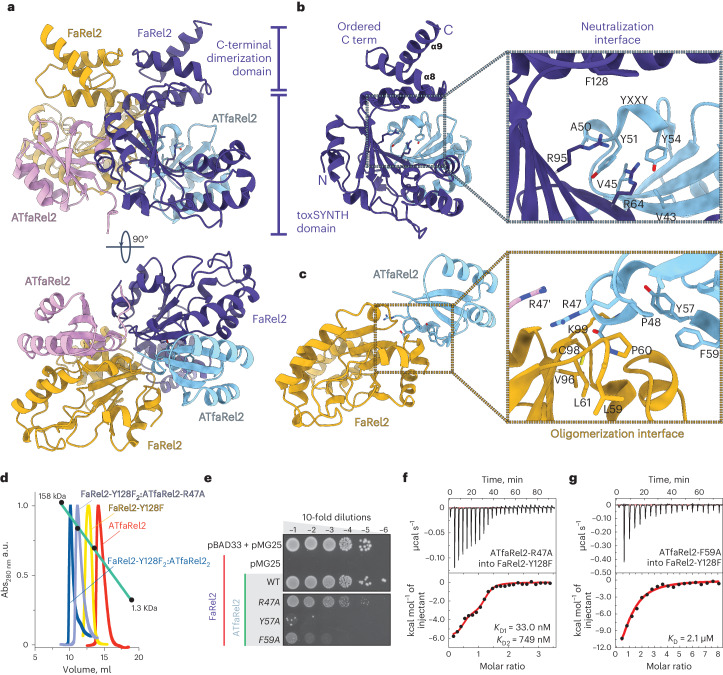
Structure of the ATfaRel2:FaRel2 complex. **a**, Structure of the heterotetrametic ATfaRel2_2_:FaRel2_2_ complex. Antitoxin units are colored light blue and pink, while the toxin units are colored dark blue and gold. The C-terminal region of FaRel2, disordered in the FaRel2:APCPP structure, in the TA complex folds into a small α-helical subdomain. **b**, Stabilization of the ATfaRel2:FaRel2 heterodimer by the primary complex interface. The ATP-binding catalytic sites have key structural roles in the interface. **c**, The secondary interface ATfaRel2:FaRel2 is formed at the two-fold symmetry axis of the complex. The interface comprises (1) cross-coordination of R47 from each antitoxin unit; (2) formation of an alternative hydrophobic interface between ATfaRel2 and FaRel2 (that is, different from the YXXY motif); and (3) interactions between the C-terminal α-helical regions of the two FaRel2 units. **d**, Analytical SEC of ATfaRel2_2_:FaRel2-Y128F_2_ (dark-blue trace), ATfaRel2-R47A_2_:FaRel2-Y128F_2_ (lilac), FaRel2-Y128F (yellow) and ATfaRel2 (red). **e**, Probing of the secondary FaRel2:ATfaRel2 interface through toxicity neutralization assays. Serial dilutions of *E.* *coli* strains expressing FaRel2 alone or coexpressed with ATfaRel2 (WT or R47A, Y57A and Y59A variants) were plated on solid LB medium and scored after 16 h at 37 °C. **f**,**g**, Binding of R47A-substituted (**f**) and F59A-substituted (**g**) ATfaRel2 to Y128F-substituted FaRel2, monitored by ITC.

To uncover the mechanism of toxin neutralization, we determined the structure of the ATfaRel2:FaRel2 complex. The structure reveals a ATfaRel2_2_:FaRel2_2_ heterotetrametric arrangement (Fig. 3a–c). The 2:2 stoichiometry was confirmed in solution by SEC (62 kDa versus the theoretical 68 kDa; Fig. 3d) and was consistent with the ITC measurements (Fig. 1f).

The C-terminal region of FaRel2, disordered in the FaRel2:APCPP complex, folds into an α-helical subdomain upon complex formation and provides a dimerization interface mediated only by toxin–toxin interactions. By contrast, in the bound state, the conformation of ATfaRel2 matches that of the free antitoxin, with both structures superimposing with an r.m.s.d. of 0.4 Å (Supplementary Fig. 3e). The primary interface between ATfaRel2 and FaRel2 has an area of 1,190.0 Å^2^, with ATfaRel2 sterically blocking access of the ATP substrate to the pyrophosphate donor binding site of the toxSYNTH domain (Fig. 3b). Through this large interface, ATfaRel2 contacts several key functional regions of FaRel2: (1) the long N-terminal α-helix α3 (a structural element that is often involved in allosteric crosstalk in many RSH enzymes and interacts with α1 from ATfaRel2); (2) the basic α-helix α4 (involved in the stabilization of the ATP triphosphate group, which is coordinated by ATfaRel2 through β3 and the β3–α1 loop); (3) the central β-sheet (through β1, β2 and β5, which harbor the catalytic center and adenine coordinating residues); and (4) the C-terminal cap of the α-helix α6 (which is disordered in the FaRel2:APCPP complex and folds into an α-helical dimerization region when bound to ATfaRel2). The only notable exception was the predicted tRNA recognition site that remains solvent accessible (Supplementary Fig. 3f).

The center of the neutralization interface is formed by the antiparallel β-strand interaction between FaRel2 β1 and ATfaRel2 β3 that connects the β-sheets of both proteins, extending the core of the complex. Residues I42–T46 of β3 form multiple van der Waals contacts with FaRel2 β1, further stabilizing the complex. Residues V43 and I45 serve as a scaffold, orienting the YXXY 3_10_-helix that anchors ATfaRel2 (Fig. 3b). As predicted for pZFD^CapRel^ (ref. ^10^), this hydrophobic tether projects Y51 and Y54 into the ATP-binding site through a *π*-stacking arrangement with R64 and R95, which precludes adenine coordination to the pyrophosphate donor site of FaRel2. These results suggest that, while this mechanism of neutralization is likely the same at the structural level to that proposed for CapRel^SJ46^, the energetics of neutralization are certainly different, with the dynamic association of pZFD^CapRel^ regulating toxSYNTH^CapRel^ in cis, contrasting with the stable in trans neutralization in the ATfaRel2:FaRel2 complex. These differences could have important implications for triggering these systems.

### Dimerization enhances toxin neutralization by ATfaRel2

Compared with pZFD^CapRel^, ATfaRel2 is considerably more tolerant to substitutions in the toxin-binding interface (compare Fig. 1e with Extended Data Fig. 3j in ref. ^10^). The structure of the ATfaRel2_2_:FaRel2_2_ complex reveals that ATfaRel2 engages the neighboring FaRel2 in the heterotetramer through a secondary interface that is half the size of the primary one (~550.0 Å^2^ versus 1,190.0 Å^2^), thus effectively crosslinking the complex (Supplementary Fig. 4a–c). We hypothesized that the stable oligomeric nature of ATfaRel2_2_:FaRel2_2_ compensates for the lack of TA colocalization enforced in the monomeric CapRel^SJ46^ through fusion of the toxin and antitoxin domains into one polypeptide. On this basis, substitutions disrupting the ATfaRel2_2_:FaRel2_2_ oligomerization interface (and not affecting the primary neutralization interface at the active site) would compromise the TA recognition.

To test this hypothesis, we subjected the secondary interface to single-residue substitutions and assessed the complex stability in vivo through toxicity neutralization assays (Fig. 3e). The three targeted residues R47A, Y57A and F59A of ATfaRel2 are all distant from the main contact interface that blocks access of ATP to the active site (Fig. 3c). R47 is located on the two-fold symmetry axis of the complex and the side chains of R47 from each ATfaRel2 interlock through *π*–*π* interactions. Y57 and F59 are part of a small hydrophobic core that defines the secondary oligomerization interface. The R47A substitution resulted in a modest defect, whereas Y57A and F59A compromised the neutralization severely (Fig. 3d).

The direct interrogation of these interactions by ITC was in good agreement with the in vivo data. The R47A substitution efficiently perturbs the secondary interface and decouples the highly cooperative tetramer formation observed in the WT protein (Fig. 3f and Supplementary Table 3). The first high-affinity binding event (*K*_D_ = 35 nM with a stoichiometry of 0.4) followed a lower-affinity recognition event (*K*_D_ = 750 nM with a stoichiometry of 0.8). This likely represents the initial neutralization of FaRel2 (with a 2:1 TA ratio) followed by the formation of a less stable 2:2 tetramer. The impact of F59A on the affinity was even stronger, with a 60-fold decrease in affinity and a confirmed 1:1 binding molar ratio, indicating an interaction mediated by only the primary interface (Fig. 3g and Supplementary Table 3). These results were consistent with SEC experiments that revealed a decrease in size of the TA complex from the estimated 70.8 kDa of the WT (consistent with a 2:2 A:T stoichiometry) to 51.3 kDa suggestive of a ATfaRel2-R47A:FaRel2-Y128F_2_ complex (1:2 A:T stoichiometry) (Fig. 3d and Supplementary Table 2). The observation of a stable ATfaRel2-R47A:FaRel2-Y128F_2_ complex in SEC matched the high-affinity interaction observed by ITC with ATfaRel2-R47A (Supplementary Table 2). It is, thus, likely that the C-terminal region of FaRel2 that folds upon TA complex formation and provides a large FaRel2:FaRel2 interface in the complex is still capable of partially stabilizing the oligomer against the effect of mild substitutions such as R47A but not against F59A, which had a major effect on complex formation.

### FaRel2 C terminus stabilizes the heterotetrameric complex

Upon formation of the ATfaRel2_2_:FaRel2_2_ complex, the C-terminal regions of the two individual FaRel2 toxin polypeptides fold into a dimerization region with four α-helices that contributes 670 Å^2^ to the TA interface (Supplementary Fig. 4d). This folding upon binding interaction likely has an important role in the overall stability of the heterotetramer. Guided by the structure of FaRel2 bound to APCPP, we constructed a truncated version of FaRel2 (FaRel2-Δ166–206) lacking the C-terminal disordered part. FaRel2-Δ166–206 interacted with ATfaRel2 with a *K*_D_ of 3.3 μM, as measured by ITC (Supplementary Fig. 4e). This ~90-fold drop in affinity of FaRel2-Δ166–206 for ATfaRel2 underscores the strong contribution of oligomerization to the overall energetics of complex formation. In the case of the WT TA complex, the binding was both entropically and enthalpically driven. The entropic penalty from the folding of the FaRel2 C terminus was likely compensated for by the configurational entropy associated with the large hydrophobic surface buried upon binding (Supplementary Table 3). Together with the strong enthalpic component that accompanied the oligomerization through the C-terminal α-helical region, this resulted in very stable heterotetramerization. Because the FaRel2-Δ166–206 truncation removes the enthalpic contribution of the C terminus, binding to ATfaRel2 was, as expected, predominantly entropically driven, resulting in a less stable complex (Supplementary Table 3). Collectively, these results suggest that, while the main TA interface drives toxin neutralization, the oligomerization further stabilizes the interaction between ATfaRel2 and the toxSYNTH domain of FaRel2. Additional contacts of the main interface YXXY motif with the folded C-terminal α-helical FaRel2:FaRel2 interface link the oligomerization with toxin neutralization, hinting at a potential allosteric path for activation and toxin release.

### tRNA-pyrophosphorylating toxins specifically bind tRNA

**Fig. 4:**
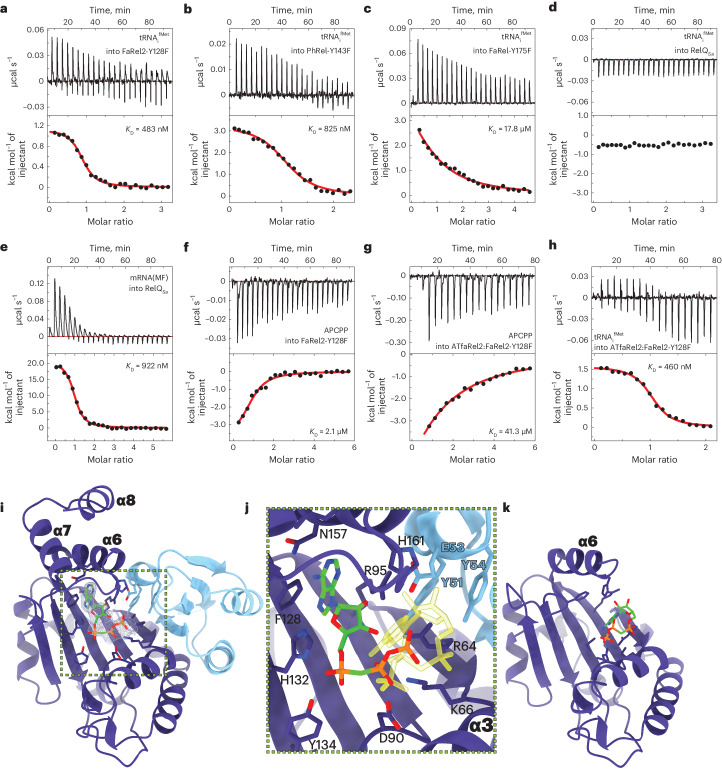
Energetic and structural basis of substrate recognition by toxSASs and non-toxSASs. **a**–**d**, Binding of FaRel2-Y128F (**a**), PhRel2-Y143F (**b**), FaRel-Y175F (**c**) and WT RelQ_*Sa*_ (**d**) to deacylated initiator tRNA_i_^fMet^, monitored by ITC. **e**, Binding of mRNA(MF) to WT RelQ_*Sa*_, monitored by ITC. **f**,**g**, Binding of APCPP to FaRel2-Y128F (**f**) and ATfaRel2_2_:FaRel2-Y128F_2_ complex (**g**), monitored by ITC. **h**, Binding of tRNA_i_^fMet^ to the ATfaRel2_2_:FaRel2-Y128F_2_ complex, monitored by ITC. **i**, Structure of ATfaRel2_2_:FaRel2-Y128F_2_ bound to APCPP (green). The unbiased mFo-DFc electron density map corresponding to the bound APCPP is shown in gray. **j**, Details of the coordination of APCPP (green) in the acceptor site when bound to ATfaRel2_2_:FaRel2-Y128F_2_. The adenosine base is coordinated by the G-loop F128 and the 5′ β and γ phosphates extend to the basic patch of α4. There, they bind in a reverse orientation compared with APCPP in the FaRel2:APCPP complex, underscoring that the nucleotide is bound in a state incompatible with phosphate transfer. For comparison, the APCPP in the orientation observed in the complex with FaRel2 is shown in light yellow. Active site residues of FaRel2 are labeled in black and the residues from the YXXY motif of ATfaRel2 are labeled in light blue. **k**, FaRel2-Y128F:APCPP complex with APCPP placed in the donor site in a catalytically compatible orientation, presented in the same pose as **i**. In the absence of ATfaRel2, α7 and α8 are not visible in the electron density.

Given the low concentrations toxSAS toxins are typically found in the cell (below the detection levels of current techniques)^20,21^, tRNA-phosphorylating activity would likely depend on a strong and specific association with tRNAs. We used ITC to examine the tRNA binding capacity of a representative toxSAS and housekeeping SASs: tRNA-phosphorylating *Coprobacillus* sp. D7 FaRel2 and *Mycobacterium* phage Phrann PhRel, (pp)pApp-synthesizing *C.* *marina* FaRel and (p)ppGpp-synthesizing *Staphylococcus aureus* SAS RelQ. FaRel2 and PhRel bound deacylated initiator tRNA_i_^fMet^ with similar affinities (*K*_D_ values of 483 nM and 825 nM, respectively; Fig. 4a,b and Supplementary Table 3). (pp)pApp synthetase FaRel had a 37-fold lower affinity to tRNA_i_^fMet^ (*K*_D_ value of 17.8 µM). No tRNA_i_^fMet^ binding was observed for (p)ppGpp-producing *Enterococcus faecalis* SAS RelQ (Fig. 4c,d and Supplementary Table 3). Lastly, *E.* *faecalis* RelQ was shown to interact with a short single-stranded model mRNA(MF) coding for MF dipeptide^22^. Our ITC experiments demonstrated that *S.* *aureus* RelQ similarly bound mRNA(MF) with submicromolar affinity (*K*_D_ value of 922 nM) (Fig. 4e and Supplementary Table 3).

### ATfaRel2 interferes with APCPP binding but not tRNA recognition

While association with ATfaRel2 decreased the affinity (*K*_D_) to APCPP ~20-fold, from 2.1 to 41.3 μM (Fig. 4f,g and Supplementary Table 3), the affinity to deacylated tRNA_i_^fMet^ was virtually the same for the free toxin and inactive TA complex (*K*_D_ value of 483 nM versus 460 nM) (Fig. 4h and Supplementary Table 3). In the cases of both free monomeric FaRel2 and the heterotetrametric ATfaRel2:FaRel2 complex, the tRNA binding had 1:1 stoichiometry with respect to FaRel2.

To further investigate the effect of AtfaRel2 binding on the interaction of FaRel2 with ATP, we determined the structure of the ATfaRel2:FaRel2 complex bound to APCPP (Fig. 4i). The high protein concentration intrinsic of the crystal lattice combined with high nucleotide concentration used for soaking facilitated the binding of APCPP to a partially blocked active site. As predicted from the structure of ATfaRel2:FaRel2, the coordination sites for the adenine, ribose and α phosphate groups at the pyrophosphate donor site are blocked by ATfaRel2. Thus, APCPP is bound in the pyrophosphate acceptor site in a conformation incompatible with pyrophosphate transfer (Fig. 4j). The adenine base is coordinated by Y128 and R95, resembling the expected coordination of the terminal adenine of the CCA tRNA moiety. The β and γ phosphates further anchor the nucleotide; however, they are observed in a reversed orientation compared with the FaRel2:APCPP complex (Fig. 4j–k).

It is instructive to compare the local charge distributions in the active sites of tRNA-modifying FaRel2 to those of (pp)pGpp and (p)ppApp alarmone synthetases Rel^13^, RelP^15^ and Tas1 (ref. ^6^). All alarmone synthetases have a large positive patch that accommodates diphosphate and triphosphate nucleotide substrates, located on the acceptor site close to the conserved Y residue that interacts with the acceptor base (Supplementary Fig. 4f–h). This positive patch is considerably smaller in FaRel2 (Supplementary Fig. 4i), which explains the misorientation of the β and γ phosphates of APCPP bound in the acceptor site of FaRel2 and the lack of alarmone synthetase activity of the tRNA-targeting toxSAS.

Collectively, our results demonstrate that FaRel2 is neutralized by the ATfaRel2 antitoxin by compromising the accommodation of ATP in the toxSYNTH active site without affecting the interaction with uncharged tRNAs. This suggests that the ATfaRel2_2_:FaRel2_2_:tRNA_2_ ternary complex could be preformed in the cell, with the toxSAS neutralized in the complex until activation is triggered.

### toxSAS neutralization is defined by catalytic activity

**Fig. 5:**
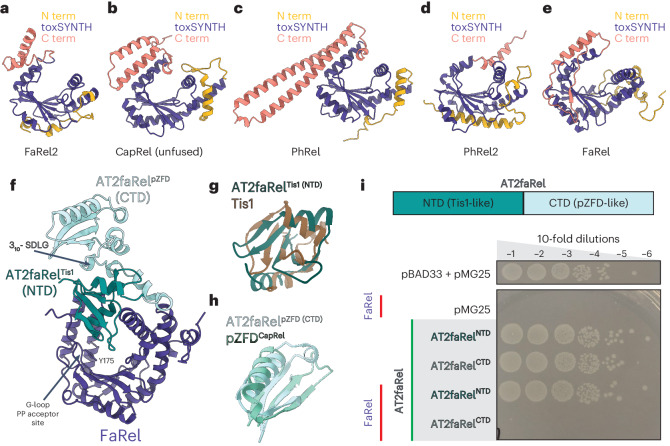
Neutralization of (pp)pApp-producing FaRel toxSAS. **a**–**f**, AlphaFold2-generated structural models of *Coprobacillus* sp. D7 FaRel2 (**a**), *M.* *tuberculosis* CapRel (unfused) (**b**), *M.* *tuberculosis* PhRel (**c**), bacteriophage Lily PhRel2 (**d**) and *C. marina* FaRel (**e**) and the AT2faRel:FaRel complex (**f**). The Tis1-like NTD of AT2faRel is colored dark green and the pZFD/ATfaRel2-like CTD is colored light cyan. AlphaFold2 predicts that FaRel is neutralized by the Tis1-like domain through the pyrophosphate acceptor site. **g**,**h**, Structural superposition of the Tis1-like NTD of AT2faRel (dark green) on Tis1 (PDB 6OX6), colored brown (**g**) and the CTD (light cyan) on pZFD^CapRel^ (PDB 7ZTB), colored light teal (**h**). **i**, Probing of the secondary FaRel2:ATfaRel2 interface through toxicity neutralization assays. Serial dilutions of *E.* *coli* strains expressing either AT2faRel^NTD^ or AT2faRel^CTD^ alone or together with FaRel were plated on solid LB medium and scored after 16 h at 37 °C.

Prompted by the conceptual differences between Tis1-mediated neutralization of Tas1 and ATfaRel2-mediated neutralization of FaRel2, we next used AlphaFold2^23^ to explore the general principles underlying the mechanisms of toxin neutralization across known toxSAS functional diversity (Fig. 5 and Supplementary Fig. 5a–l). On the toxSAS toxin side, AlphaFold2 predicts a strong conservation of a core toxSYNTH fold decorated with a variety of insertions at the N and C termini (Fig. 5a–e). On the antitoxin side, the pZFD fold is found as either a standalone neutralizing domain or part of multidomain antitoxins combined with either Tis1 or Tis1-like (Fig. 5f–h) or PanA domains^11,24^ (Supplementary Fig. 5a–j).

Structural predictions of the different neutralized complexes uncovered a general trend. Translation-targeting tRNA-pyrophosphorylating toxSASs such as fused and split CapRel, FaRel2, PhRel and PhRel2 are inhibited through the pyrophosphate donor site (Supplementary Fig. 5a–j). Conversely, metabolism-targeting (pp)pApp-producing toxSASs such as FaRel and Tas1 are neutralized through the pyrophosphate acceptor site (Fig. 5f and Supplementary Fig. 5k,l). The generality of this observation holds even in cases of multidomain antitoxins. In the case of the translation-targeting PhRel2 of bacteriophage Lily and *Bacillus subtilis* Ia1a. These toxins are neutralized by multidomain antitoxins that contain a pZFD fold subtype PAD1 (panacea-associated domain 1)^11^. However, ATphRel2 antitoxins neutralize the toxins analogously to the pZFD-mediated neutralization of CapRel and FaRel2 (Supplementary Fig. 5f,g)^24^. By contrast, the (pp)pApp-producing FaRel is inhibited by the Tis1-like domain of AT2faRel in a manner analogous to Tis1-mediated inhibition of Tas1 (Fig. 5f and Supplementary Fig. 5k,l).

We validated the structural predictions of Alphafold2 through mutagenesis and toxicity neutralization assays. In the case of PhRel2, as we showed previously^24^, the isolated PAD1 domain of *B.* *subtilis* Ia1a ATphRel2 is sufficient to neutralize the toxin. In the case of FaRel:AT2faRel, the Tis1-like N-terminal domain (NTD) of AT2faRel (Fig. 5g) is sufficient to neutralize the (pp)pApp synthetase FaRel, while the pZFD C-terminal domain (CTD; Fig. 5h) has no neutralizing activity (Fig. 5i). Interestingly, this loss of neutralizing activity by the CTD of AT2faRel is accompanied by the loss of the YXXY recognition motif of the pZFD (Fig. 5f). Collectively, our results suggest a coupling between the substrate specificity of toxSASs and the mechanism of neutralization.

## Discussion

**Fig. 6:**
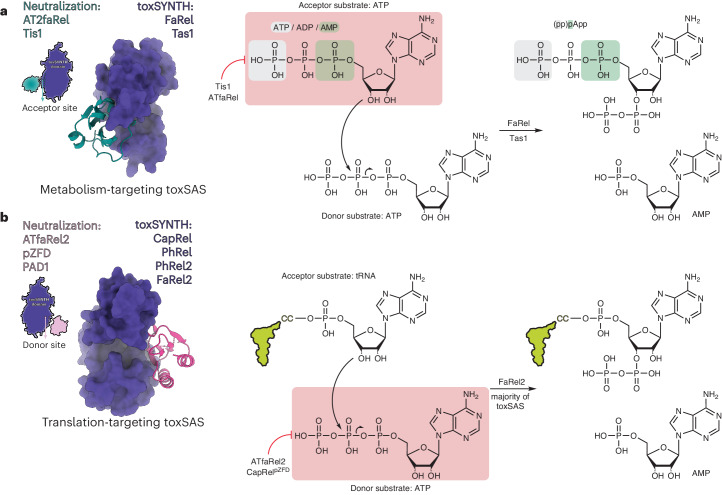
The substrate specificity of toxSASs is coupled to neutralization mechanism. Metabolism-targeting and translation-targeting toxSAS are inhibited by different strategies. **a**, Enzymatic activity of metabolism-targeting toxSASs is suppressed by inhibition of the recruitment of the pyrophosphate acceptor substrate. **b**, Translation-targeting toxSASs are neutralized by inhibition of the recruitment of the pyrophosphate donor, while binding of the pyrophosphate acceptor substrate, tRNA, is permitted. This strategy is analogous to that used for the regulation of long (p)ppGpp-producing RSHs Rel, RelA and SpoT; while the affinity to the GTP or GDP substrate nucleotide is constitutive, binding of the ATP nucleotide to the pyrophosphate donor site is under strict allosteric control.

Our current mechanistic understanding of toxSYNTH inhibition by immunity proteins and antitoxins is largely based on two landmark studies. The first study involves the structure of the neutralized complex between a monomeric (p)ppApp-producing toxSAS Tas1 and its immunity protein Tis1 (ref. ^6^). Ahmad and colleagues proposed that the enzymatic activity of Tas1 was suppressed by Tis1 through distortion of the acceptor nucleotide-binding site of toxSYNTH. The second study involves the structure of the tRNA-pyrophosphorylating toxSAS CapRel^SJ46^ (ref. ^10^), a fused monocistronic TA, with the antitoxin part comprising two anchor regions and a pZFD. In this structure, CapRel^SJ46^ appears in a catalytically competent state (that is not autoinhibited by the pZFD), as the antitoxin domain was in a conformation compatible with the enzymatic activity of toxSYNTH. Further exploration of the structural dynamics of CapRel^SJ46^ with AlphaFold2 (ref. ^23^) predicted a possible mechanism of autoinhibition mediated by the pZFD blocking the donor nucleotide-binding site of toxSYNTH (Fig. 6a,b).

Our study provides detailed mechanistic understanding of toxSAS regulation and allows generalizing the observations previously made with CapRel^SJ46^ and Tas1:Tis1 to the whole toxSAS superfamily. We put forward a model that couples toxSAS substrate specificity to the inhibition strategies used by antitoxins and immunity proteins (Fig. 6a,b). Type II TA modules are notoriously evolutionarily promiscuous, with members of the same toxin family being neutralized by unrelated antitoxins through different mechanisms^24–26^. toxSAS TAs display a strikingly clear-cut dichotomy of neutralization mechanisms. Metabolism-targeting (pp)pApp synthetases such as Tas1 and FaRel are inhibited by Tis1-like antitoxins through the occlusion of the pyrophosphate acceptor nucleotide-binding site^6^ (Fig. 6a). Conversely, translation-targeting tRNA pyrophosphotransferases such as FaRel2 and CapRel are inhibited by pZFD interfering with the binding site for pyrophosphate donor ATP (Fig. 6b), which echoes the regulatory strategy used in the autoinhibition of multidomain ‘long’ (p)ppGpp-synthesizing housekeeping RSHs^12,27^. While the antitoxins and immunity proteins mediating the two neutralization strategies have diverged in sequence, the neutralizing elements display strong structural conservation.

Type II TA antitoxins often rely on unstructured elements and disordered domains that fold upon binding for toxin neutralization^25,28–34^. In many cases, the structural plasticity of the disordered region of antitoxins allows them to couple toxin neutralization with transcriptional autoregulation^29,35,36^ to balance the cellular T:A ratio. Interestingly, this feature of TA regulation seems so prevalent that, in the exception provided by the GraTA system (from the RelBE superfamily), which contains a well-folded and globular antitoxin, the disordered region leaps to the toxin GraT retaining a role as a crucial regulatory element^37^. The structurally defined lock-and-key neutralization specificity of toxSASs is, thus, uncommon and may underlie their ‘switch’ nature. In this sense, it is instructive to compare the structural–energetic interplay of the fused CapRel^SJ46^ TA with the bipartite ATfaRel2:FaRel2 TA. The forced colocalization of the toxin and antitoxin elements of CapRel^SJ46^ is compatible with a conformationally dynamic enzyme and offsets the entropic penalty associated with the antitoxin assuming a compact and structured neutralizing state (Supplementary Fig. 6a). These intrinsic dynamics facilitate the formation of the CapRel^SJ46^:Gp57 complex that triggers the enzyme. By contrast, in the bipartite ATfaRel2:FaRel2 TA system, the free antitoxin naturally assumes the optimal conformation for neutralization. The entropic penalty associated with FaRel2 folding is compensated by the formation of the heterotetramer and a tight TA complex. Thus, the loss of colocalization is compensated by the stabilizing effect of oligomerization. Our findings suggest that offsetting this oligomeric structure could be the key to triggering these bipartite systems (Supplementary Fig. 6b).

ZFDs perform various molecular recognition functions^38^ facilitated by a marked structural plasticity^39^. Structurally related pZFDs mediate both phage recognition and toxin autoinhibition in fused tRNA-pyrophosphorylating TA CapRel^SJ46^ (ref. ^10^). PAD1 has the same fold as pZFD and directly mediates the neutralization of *B.* *subtilis* Ia1a and *Clostridium hylemonae* the tRNA-pyrophosphorylating PhRel2 toxSAS^11,24^. Conversely, the pZFD-like CTD of AT2faRel is not essential for the inhibition of the (pp)pApp synthetase FaRel, which is neutralized by its Tis1-like NTD (Fig. 5c,b). This suggests that the pZFD-like domain of AT2faRel performs a different sensory function. This evolutionary dynamic is reminiscent of PanA^11^ and HigA^37,40^ antitoxins. While, in the case of single-domain antitoxins, PanA directly mediates toxin neutralization, in multidomain antitoxins, PanA domains are not the direct neutralization element; instead, these domains were hypothesized to sense the TA-activating triggers^24^. Establishing the putative sensory functions of pZFDs in translation-targeting and metabolism-targeting toxSASs is one of the remaining challenges in the field.

## Methods

### Plasmid construction

Fragments of WT *aTfaRel2* and its substituted variants (V43A, I45A, A50M, Y51A and Y54A) were PCR-amplified with primers VTK198 and VTK199 and templates VHp278 (WT), VHp1225 (V43A), VHp1226 (I45A), VHp1227 (A50M), VHp1236 (Y51A) or VHp1228 (Y54A). Using Gibson assembly, the resulting linear DNA fragment was inserted into linearized pMG25 using pMG HiFi For and pMG HiFi Rev primers.

### Sequence analysis

Representative FaRel2 and cognate ATFaRel2 sequences were retrieved using webFlaGs^41^, implementing the protein basic local alignment search tool (BLASTp) in the National Center for Biotechnology Information (NCBI) RefSeq Select database and otherwise default settings. Sequences were aligned using MAFFT version 7.490 with the L-INS-i strategy^42^.

### Toxicity neutralization assays

The experiments were performed as described previously^7^. The assays were performed on Luria–Bertani (LB) medium plates (BD). We used the *Escherichia coli* BW25113 strain cotransformed with two different plasmid systems for controllable expression of toxins and antitoxins. We used a pair of compatible plasmids: pMG25 for antitoxin expression (high copy number, ColE1 origin of replication (pUC), Amp^R^, antitoxin expressed under the control of IPTG-inducible P_A1/04/03_ promoter^43^] and pBAD33 for toxin expression (medium copy number, p15A origin of replication, Cml^R^, toxins expressed under the control of arabinose-inducible P_BAD_ promoter^44^). The cells were grown in liquid LB medium (BD) supplemented with 0.2% glucose (repression conditions), 100 µg ml^−1^ ampicillin (AppliChem) and 20 µg ml^−1^ chloramphenicol (AppliChem). Serial dilutions were spotted on solid LB plates supplemented with 0.2% arabinose, as well as 100 µg ml^−1^ ampicillin (AppliChem) and 20 µg ml^−1^ chloramphenicol (AppliChem), and bacterial growth was scored after 16-h incubation at 37 °C.

### Protein purification

ATfaRel2, Farel2-Y128F and the different variants of the proteins were expressed in *E.* *coli* BL21DE3. The proteins were produced with a His_6_ tag at the N terminus, followed by a tobacco etch virus (TEV) protease cleavage site for ATfaRel2 and the different ATfaRel2 variants and a SUMO (small ubiquitin-like modifier) tag for FaRel2-Y128F. Cultures were grown in LB medium supplemented with kanamycin (50 μg ml^−1^) at 37 °C with aeration. Expression was induced with 0.5 mM IPTG when the cells carrying the plasmid reached an optical density at 600 nm (OD_600 nm_) of ~0.5–0.8. After induction, the cells were harvested 16 h later by centrifugation and resuspended with buffer (25 mM HEPES pH 7.6, 1 M NaCl, 5 mM MgCl_2_ and 1 mM TCEP) supplied with cOmplete protease inhibitor cocktail (Roche). The resuspended cells were flash-frozen in liquid nitrogen and stored at −80 °C.

The cell extracts were lysed using an Emulsiflex cell disruptor and the lysate was centrifuged to remove cell debris for 45 min at 25,000*g*. In both cases, the supernatant was loaded onto a 1-ml HiTrap Ni-NTA column (Cytiva) coupled to a fast protein liquid chromatography (FPLC) system (ÄKTA Explorer) equilibrated with buffer A (25 mM HEPES pH 7.6, 1 M NaCl, 5 mM MgCl_2_, 1 mM TCEP and 20 mM imidazole). The column was washed with a linear gradient of buffer B (25 mM HEPES pH 7.6, 1 M NaCl, 5 mM MgCl_2_, 1 mM TCEP and 500 mM imidazole). After tag removal, all individual proteins were further purified by SEC in a Superdex 75 Increase 10/30 (Cytiva) column using 25 mM HEPES pH 7.6, 300 mM NaCl, 2 mM MgCl_2_ and 1 mM TCEP. Sample purity was confirmed by SDS–PAGE. The ATfaRel2:FaRel2-Y128F complex was obtained by mixing both proteins in a 1:1.2 molar ratio with an excess of antitoxin that was separated by SEC.

### Analytical SEC

For the analytical SEC, 150 μl of each protein at a concentration of 1 mg ml^−1^ was loaded on a Superdex 300 Increase 1030 column (Cytiva) previously equilibrated in SEC buffer (25 mM HEPES pH 7.6, 200 mM NaCl, 2 mM MgCl_2_ and 1 mM TCEP). The progress of the chromatography was monitored by the OD_280_ value.

### Crystallization

Before crystallization, FaRel2-Y128F, ATfaRel2 and the ATfaRel2 variants were purified from the TEV cleavage reaction in the SEC buffer and concentrated to 8–10 mg ml^−1^. Screening of the crystallization conditions was carried out using the sitting-drop vapor diffusion method. The drops were set up in Swiss (MRC) 96-well two-drop UVP sitting-drop plates using the Mosquito HTS system (TTP Labtech). Then, 0.1-μl drops of protein and precipitant solution were equilibrated to 80 μl of precipitant solution in the reservoir. Commercially available screens LMB and SG1 (Molecular Dimensions) were used to test the crystallization conditions. The conditions resulting in diffracting crystals are listed in Supplementary Table 1. The crystals used for data collection the 0.2-μl drops used in the screens were scaled to 2-μl drops.

### Structure determination

All the data were processed with the XDS suite^45^ and scaled with Aimless^46^. In all cases, the unit cell content was estimated with the program MATTHEW COEF from the CCP4 program suite^47^. The crystals of ATfaRel2 diffracted on average to ~1.3 Å. The analysis of the crystal anisotropy by the STARANISO server (http://staraniso.globalphasing.org/) proposed a resolution of 1.24 Å (with 1.24 Å in a*, 1.33 Å in b* and 1.43 Å in c*). We used Arcimboldo_Lite^48^ to perform ab initio phasing and solved the structure of ATfaRel2 in combination with Phaser^49^ and SHELXE^50,51^. The solution contained 75 of the 99 residues; the final structure was completed by manual building using Coot^52^ and refined with Buster/TNT^53^ (*R*/*R*_free_ = 18.6/20.8).

In the case of the crystals of the FaRel2:APCPP complex, the analysis of the diffraction data suggested a resolution of 2.62 Å (with diffraction limits of 2.53 Å in a*, 2.67 Å in b* and 2.97 Å in c*) based on which we selected 2.62 Å as the resolution cut-ff. We used the coordinates of CapRel^SJ46^ (PDB 7ZTB) as the search model for the toxSYNTH domain of FaRel2 in complex with APCPP. The molecular replacement (MR) solution from Phaser^49^ was used in combination with Rosetta as implemented in the MR-Rosetta suite from the Phenix package^54^. After several iterations of manual building with Coot^52^ and maximum likelihood refinement as implemented in Buster/TNT^53^, the model was extended to cover all the residues (*R*/*R*_free_ = 19.4/25.5).

The crystals of the ATfaRel2:FaRel2 complex were obtained in the *P*2_1_2_1_2_1_ space group. The anisotropic analysis of the diffraction data suggested a resolution of 2.14 Å (with diffraction limits of 2.25 Å in a*, 3.00 Å in b* and 2.13 Å in c*). We used the refined coordinates of FaRel2 (PDB 8PU4, this work) and ATfaRel2 (PDB 8PU2, this work) as the search model for phasing and estimated the unit cell content with MATTHEW COEF from the CCP4 program suite^47^. The MR solution from Phaser^49^ was completed by manual building with Coot^52^ and refined with Buster/TNT^53^ (*R*/*R*_free_ = 18.4/23.8). To obtain the structure of the ATfaRel2:FaRel2:APCPP complex, the *P*2_1_2_1_2_1_ space group did not tolerate soaking; therefore, we grew crystals in a different condition (*F*4_1_32 space group). The coordinates of FaRel2 (PDB 8PU4, this work) and ATfaRel2 (PDB 8PU2, this work) were used for MR with Phaser^49^ and modeling was completed by manual building with Coot^52^ and refinement with Buster/TNT^53^ (*R*/*R*_free_ = 21.8/23.2). Supplementary Table 1 details all the X-ray data collection and refinement statistics.

### ITC

All titrations were performed with an Affinity ITC (TA instruments) at 25 °C. For antitoxin versus toxin titrations, ATfaRel2 and its substituted variants were loaded in the instrument syringe at 200–150 μM and FaRel2-Y128F was used in the cell at 15–20 μM. In the case of the titrations of tRNA and mRNA versus toxSASs, 150 μM tRNA or mRNA was titrated into 15 μM FaRel2-Y128F, PhRel-Y143F, FaRel-Y175F, RelQ or the ATfaRel2:FaRel2-Y128F complex. For the titrations with nucleotides, APCPP was loaded in the instrument syringe at 180 μM and FaRel2-Y128F or the ATfaRel2:FaRel2-Y128F complex was used in the cell at 15 μM. All titrations were performed in 25 mM HEPES pH 7.6, 300 mM NaCl, 2 mM MgCl_2_ and 1 mM TCEP. Final concentrations were verified by the OD_280_ value using a Nanodrop One (Thermo Fisher Scientific). All ITC measurements were performed by titrating a constant volume of 2 μl into the ITC cell using a constant stirring rate of 75 rpm. All data were processed, buffer-corrected and analyzed using the NanoAnalyse and Origin software packages. Supplementary Table 3 details all the thermodynamic parameters derived from the ITC titrations.

### Reporting summary

Further information on research design is available in the Nature Portfolio Reporting Summary linked to this article.

## Online content

Any methods, additional references, Nature Portfolio reporting summaries, source data, extended data, supplementary information, acknowledgements, peer review information; details of author contributions and competing interests; and statements of data and code availability are available at 10.1038/s41589-024-01630-4.

## Supplementary information

Supplementary Information Supplementary Figs. 1–6 and Tables 1–4.

Reporting Summary

Supplementary Data 1 Strains.

## Data Availability

The data generated in this study are provided in the Supplementary Information provided with this paper. All coordinates were deposited in the PDB under accession numbers 8PU1, 8PU2, 8PU3 and 8PU4. Data are also available from the corresponding authors upon request.
