# Supplementary material for: Mechanisms of neutralization of toxSAS from toxin–antitoxin modules
Source: Nat Chem Biol. 2024 Jun 4;21(2):182–92. doi: 10.1038/s41589-024-01630-4 (PMC11782079; doi:10.1038/s41589-024-01630-4)
Supplement: Supplementary file 1 — Supplementary Figs. 1–6 and Tables 1–4. [file 41589_2024_1630_MOESM1_ESM.pdf]

nature chemical biology

Supplementary information https://doi.org/10.1038/s41589-024-01630-4

Mechanisms of neutralization of toxSAS from toxin–antitoxin modules

In the format provided by the authors and unedited

SUPPLEMENTARY FIGURES

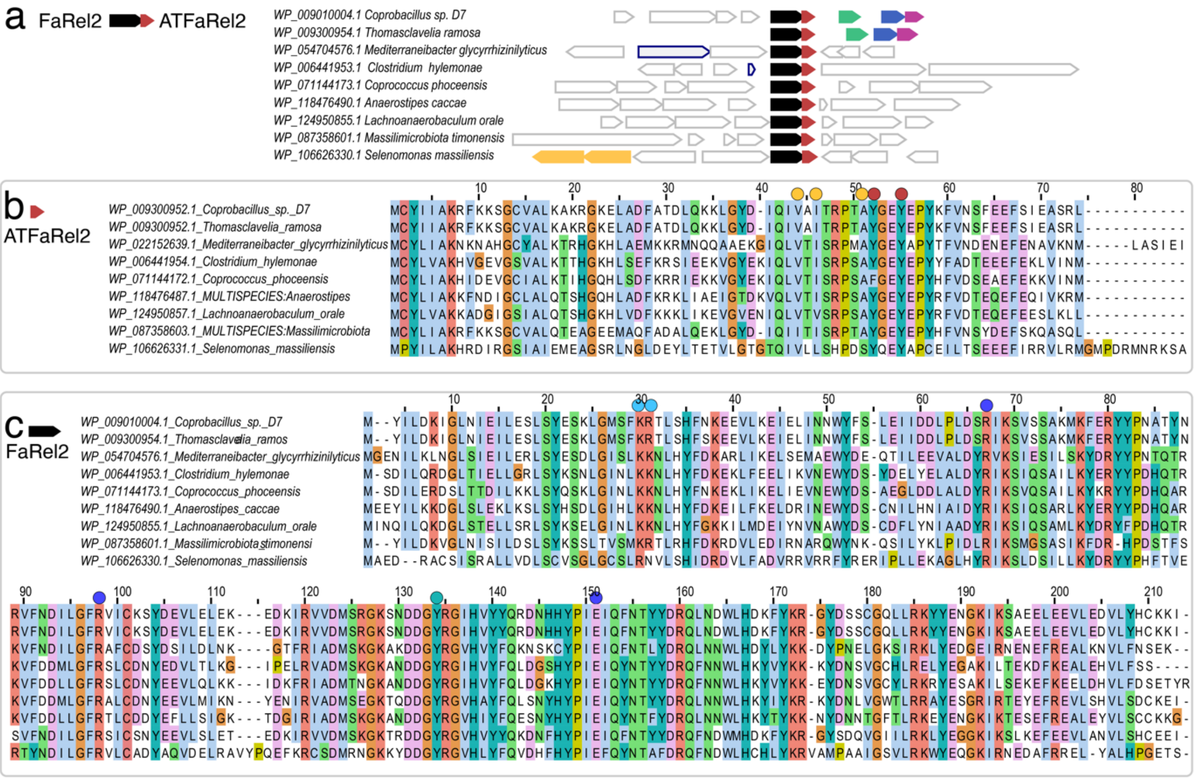

Supplementary Fig. 1. Conservation of FaRel2 and ATFaRel2. A representative set of FaRel2 and cognate ATFaRel2 sequences were identified using webFlaGs[43] **(a)**. Grey outlined genes are unconserved in the neighborhood, while colored are conserved. Pseudogenes are shown with blue outlines. Multiple sequence alignments of ATFaRel2 **(b)** and FaRel2 **(c)** show conservation of sites along the length of the proteins. Substituted sites of ATFaRel2 located withing scaffolding β1 element are indicated with orange circles and those from the YXXY motif are highlighted with red circles. Y128 from FaRel2 is indicated with a green circle. Residues K28 and R29 that as involved in tRNA recognition are indicated with a light blue circles, while the conserved active site residues R64 and R95 as well as the catalytic E154 are highlighted with dark blue circles.

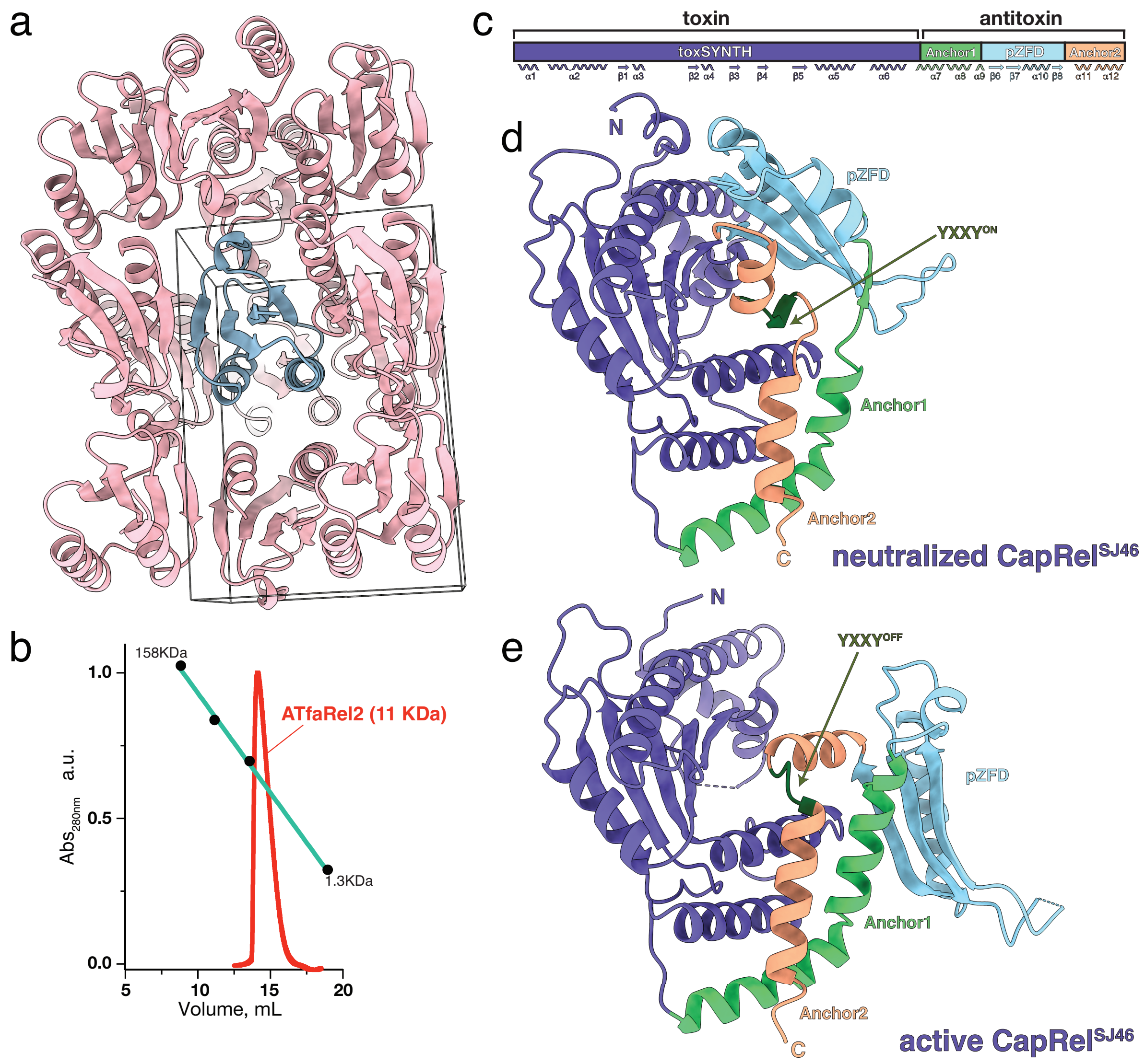

Supplementary Fig. 2. Biophysical properties of ATfaRel2.

(**a**) Unit cell content and packing of ATfaRel2 (in blue). Crystallographic symmetry mates are shown in pink. The analysis of the lattice packing suggests ATfaRel2 is a monomer. (**b**) Analytical SEC of *Coprobacillus* sp. D7 ATfaRel2 confirms the antitoxin is a monomer in solution. (**c**) Domain architecture of *Salmonella* phage SJ46 CapRel^{JS46} fused toxin-antitoxin. (**d**) AlphaFold-predicted structure of CapRel^{JS46} in catalytically inactive state[9] (**e**) crystal structure of CapRel^{JS46} in catalytically active state (PDB ID 7ZTB, Ref.[9]). In both, (d) and (e), the toxSYNTH domain is colored in dark blue, and the pZFD domain in light blue. Two α-helical substructures connect both domains, Anchor1 colored in green and Anchor2 in salmon. The open/close conformational switch region that contains the YXXY neutralization motif is colored in dark green.

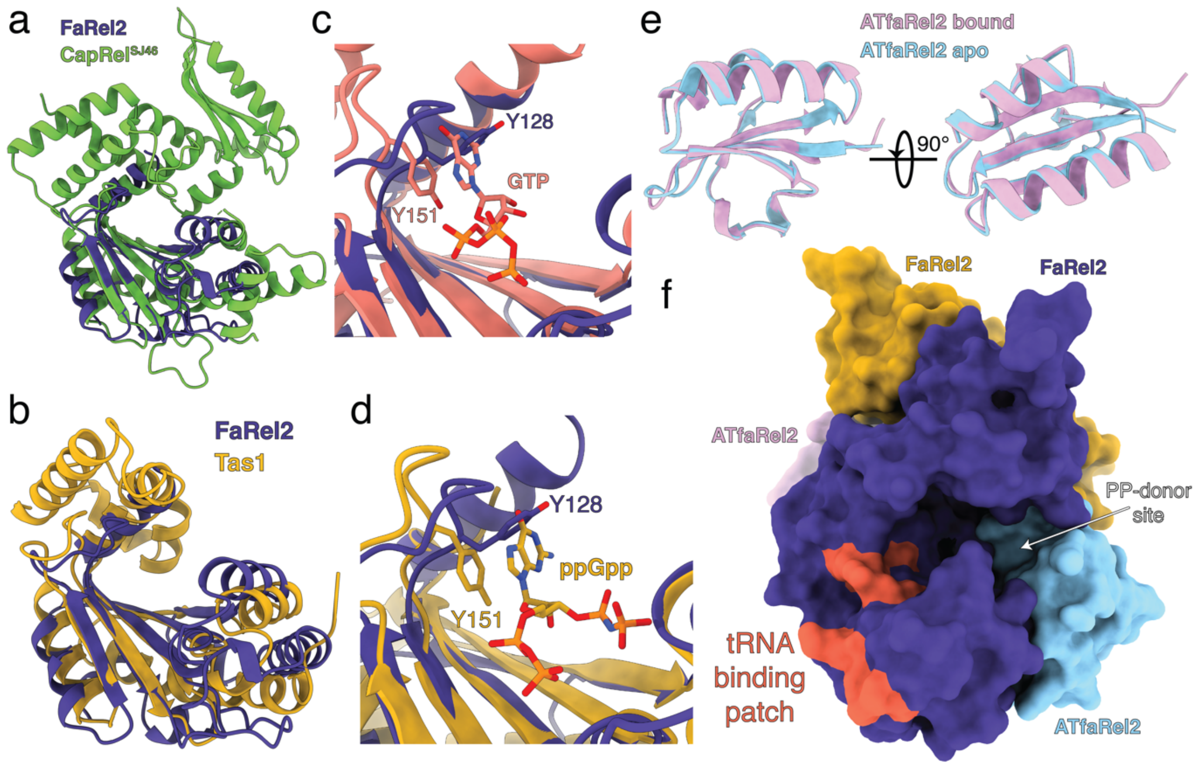

Supplementary Fig. 3. Conformational landscape of FaRel2.

(**a**) Structural alignment of *Coprobacillus* sp. D7 FaRel2 with tRNA-modifying *Salmonella* phage SJ46 CapRel^{JS46} in catalytically active state (PDB ID 7ZTB, Ref.[9]). (**b**) Structural alignment of FaRel2 with (pp)pApp-synthesising *S. aeruginosa* secretion system effector Tas1[6]. (**c**) Superposition of the active site FaRel2 (in dark blue) on the structure of the RelQ-GTP complex (in salmon). The orientation of Y128 of FaRel2 is incompatible with nucleotide binding. This mode of guanosine coordination is conserved in the SYNTH active site of SAS as shown in the comparison of FaRel2 with RelQ in complex with ppGpp (d). (**e**) Structural alignment of *Coprobacillus* sp. D7 ATfaRel2 in unbound apo-state (coloured in sky blue) and FaRel2-bound states (in pink). (**f**) Surface representation of the FaRel2:ATfaRel2 complex coloured as in **Fig. 3a**. The tRNA-binding patch that was experimentally established previously by Kurata *et al.*[7] (in red) and the ATfaRel2-binding interface are located on the opposite surfaces of the protein.

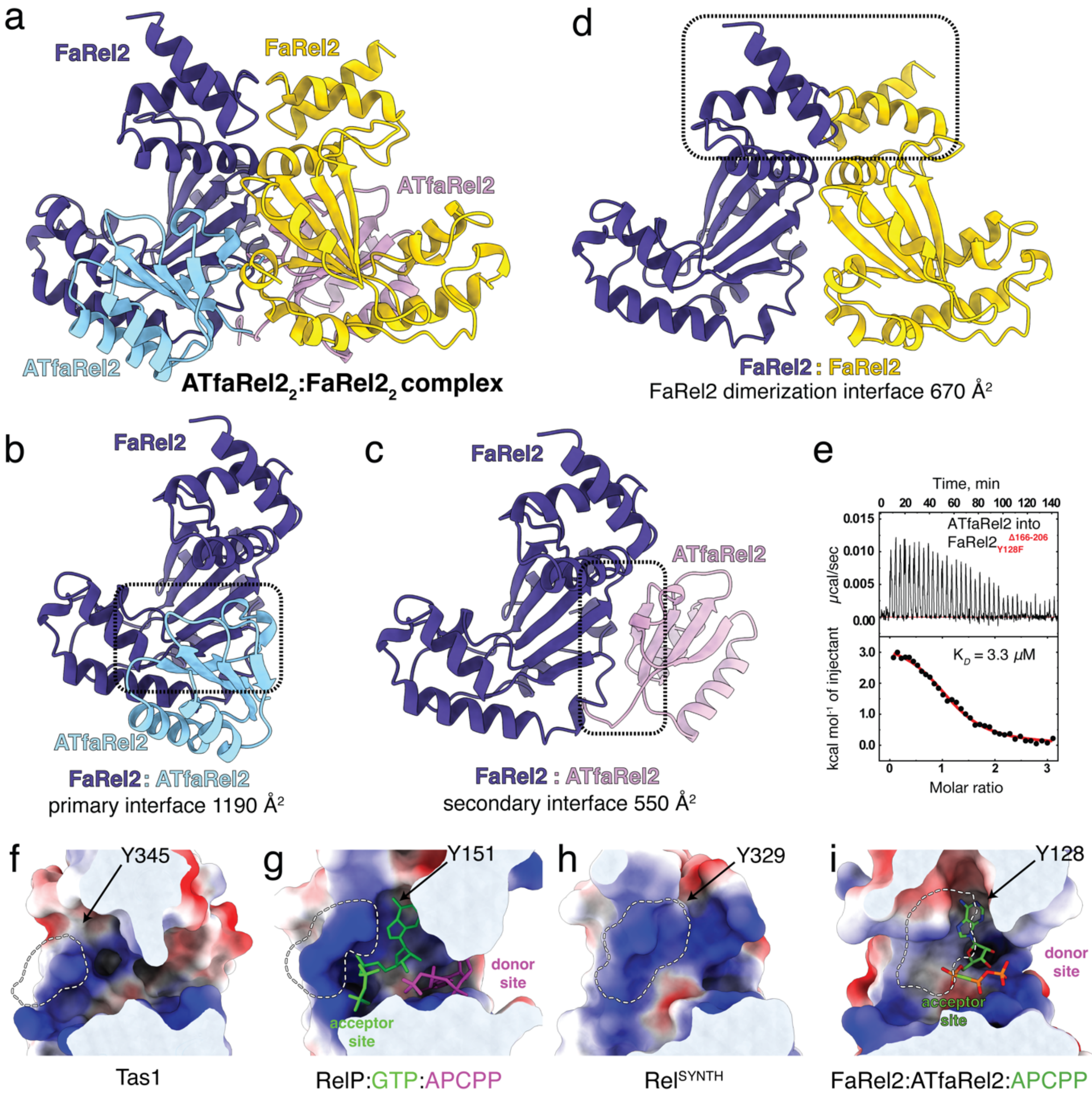

Supplementary Fig. 4. Translation–targeting toxSAS interaction interfaces.

(**a**) Structure of the ATfaRel2$_2$:FaRel2$_2$ heterotetramer, the complex is stabilised by three different interfaces. (**b**) A primary neutralization interface which involves largest contact surface. This 1190 Å^2 interaction site involves blocking the ATP binding site of the toxSYNTH. (**c**) The secondary 550 Å^2 interaction point links FaRel2 with the neighbouring ATfaRel2 (not engaged via the ATP binding site). (**d**) A third 670 Å^2 interaction interface is formed via the folding of the C-terminal region of FaRel2 upon binding to ATfaRel2 (bottom left). This interface defines a FaRel2 dimer within the heterotetramer. (**e**) ITC titration of ATfaRel2 into FaRel2$^{Y128F\ \Delta 166\text{-}206}$, a truncated variant of FaRel2 that lacks the C-terminal disordered domain. (**f**) SYNTH active site of Tas1[6], the G-loop Y345 is highlighted with an

arrow. (**g**) SYNTH active site of RelP in a pre-catalysis state bound to GTP and APCPP. The G-loop Y151 is indicated with a black arrow. (**h**) SYNTH active site of *T. thermophilus* Rel. The G-loop Y329 is indicated with a black arrow (Ref.[19]). (**i**) toxSYNTH active site of *Coprobacillus* sp. D7 FaRel2 in complex with APCPP. The G-loop Y128 is indicated with a black arrow. On all the panels the surface patch neighboring the conserved Tyr is contoured by a dashed white line. This patch is strongly positively charged in alarmone-synthesising SAS/toxSAS/long RSHs (a-c) for efficient coordination of di- and tri-phosphate nucleotide substrates. In the case of FaRel2 (d) this region overlaps with the tRNA-binding patch and is not as electropositive. This likely reflects the relatively less charged phosphodiester tRNA backbone that is accommodated by FaRel2's active site.

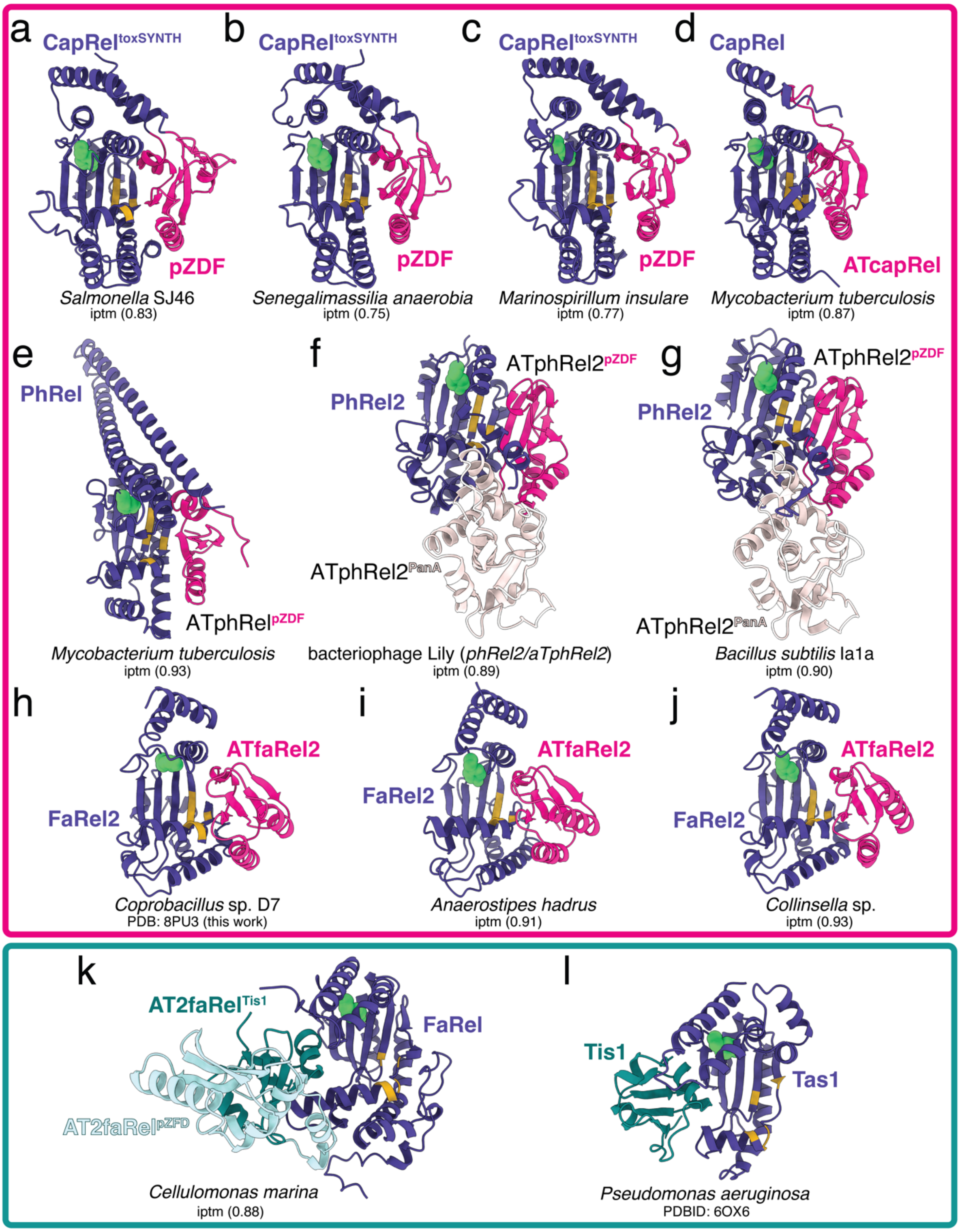

Supplementary Fig. 5. Correlation between toxSYNTH neutralisation strategy and substrate specificity.

Experimentally determined and AlphaFold2-predicted structures of toxSASs from TA complexes, fused TAs and Type IV secretion system effectors. **(a)** Neutralised CapRels including from *Salmonella* phage SJ46 CapRel^{SJ46} fused TA. (**b-d**) ATcapRel:CapRel TA

systems from *S. anaerobia* (b), *M. insulare* (c) and *M. tuberculosis* (d), (**e**) ATphRel:PhRel from *M. tuberculosis*. (**f,g**) ATphRel2:PhRel2 from bacteriophage Lily (f) and *B. subtilis* Ia1a (g). In both cases, the ATphRel2 multidomain antitoxin is composed of an N-terminal pZFD domain (referred to as PAD1 when associated to PanA) and a C-terminal PanA domain. (**h-j**) ATfaRel2:FaRel2 from *Coprobacillus* sp. D7 (X-ray structure, this work) (h), *A. hudrus* (i) and *Collinsella* sp. (j). (**k**) *C. marina* AT2faRel:FaRel. (**l**) *P. aeruginosa* neutralised secretion system effector complex Tis1:Tas1 (X-ray structure, PDBID 6OX6, Ref.[6]). Alphafold2 interface pTM scores (iptm) are shown in the figure. In every case the PP-donor site is colored in yellow and the PP-acceptor site (represented by the conserved Y from the G-loop shown as spheres) is colored green.

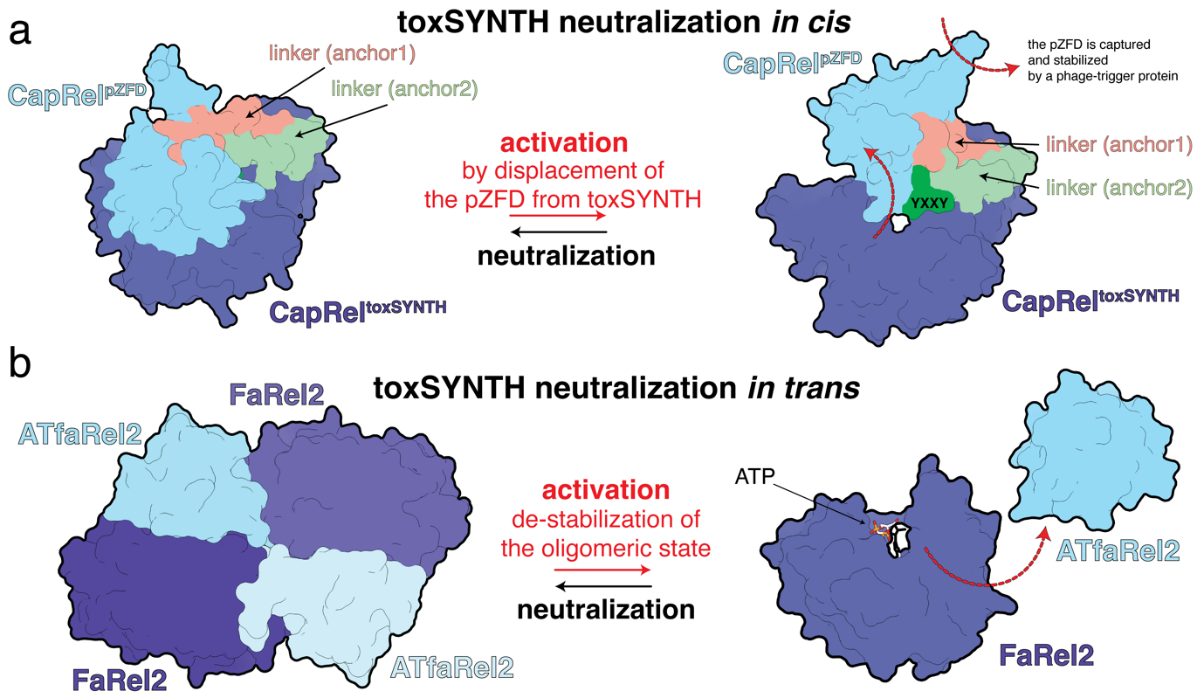

Supplementary Fig. 6. Interplay between activation and neutralization in fused systems such as CapRel vs bipartite systems such as FaRel2:ATfaRel2.

(**a**). Neutralization in CapRel occurs *in cis*, typically a phage trigger captures the pZFD precluding the inhibitory effect on the toxSYNTH domain. (**b**) By contrast in bipartite system the neutralization of the toxin occurs *in trans* stabilized by the formation of the heterotetrameric complex.

SUPPLEMENTARY TABLES

Supplementary Table 1. X-ray data collection and processing. The $CC_{1/2}$ criterion was used to determine the resolution range. Values for the outer shell are given in parentheses.

	**ATfaRel2**	**ATfaRel2$_2$-FaRel2$_2$ complex**	**FaRel2-APCPP**	**ATfaRel2$_2$-FaRel2$_2$ - APCPP complex**
Crystallization condition	15 % PEG 2000 MME, Bis Tris Propane pH 6.9, Temperature 293°C	35% v/v 1,4-Dioxane, Temperature 293°C	12 % w/v PEG 2000, 50 mM MES, pH 5.5, Temperature 277°C	3% v/v 2-Propanol and 0.1 M Sodium Chloride, pH 6.5, Temperature 293°C
Diffraction source	Soleil (PX1)	DLS (id24)	Soleil (PX2)	Soleil (PX2)
Wavelength (Å)	0.9801	0.9795	1.0000	0.9801
Temperature (K)	90.0	90.0	90.0	90.0
Detector	Eiger	Eiger	Eiger	Eiger
Detector distance (mm)	255.7	289.3	322.9	215.8
Space group	P2_12_12	P$2_12_12_1$	P2_1	F4_132
a, b, c (Å)	53.3, 34.3, 37.6	51.7, 106.2, 135.1	31.5, 60.6, 177.2	227.8, 227.8, 227.8
α, β, γ (°)	90.0, 90.0, 90.0	90.0 90.0 90.0	90.0, 90.6, 90.0	90.0 90.0 90.0
Resolution range (Å)	30.52 - 1.24 (1.37 - 1.24)	80.4 – 2.14 (3.36 – 2.14)	59.10 – 2.62 (2.83 -2.62)	46.5 - 1.70 (1.73 - 1.70)
Total No. of reflections	191338 (5867)	1074368 (52414)	220686 (8909)	3808667 (78533)
No. of unique reflections	15171 (759)	26913 (1346)	16332 (818)	55660 (2727)
Completeness (%)	92.6 (46.5)	94.1 (64.3)	90.9 (40.6)	99.95 (99.60)
Redundancy	12.6 (7.7)	39.9 (38.9)	13.5 (10.9)	68.4 (28.7)
⟨ I/σ(I)⟩	13.0 (1.3)	11.3 (1.5)	7.3 (1.4)	24.47 (0.49)
R_{merge}	0.087 (1.205)	0.33 (4.77)	0.446 (2.730)	0.196 (4.99)
CC1/2	0.998 (0.711)	0.99 (0.66)	0.985 (0.332)	1 (0.399)
Overall *B* factor / Wilson plot (Å^2)	17.18	40.97	67.37	36.80
R-factor (%)	18.69	18.37	19.44	21.8
R_{free}-factor (%)	20.85	23.83	25.54	23.2
Ramachandran profile (%)				
Core	98.61	98.00	96.37	97.8
Allowed	1.39	2.00	3.42	2.2
Outliers	0.0	0.0	0.21	0.0
R.m.s. deviations				
Bond lengths (Å)	0.06	0.013	0.01	0.012
Bond angles (°)	0.88	1.49	1.17	1.44
Number of atoms	712	4830	4230	2683
Macromolecules	596	4609	4035	2237
Solvent	111	221	99	345
Other			96	101
B-factors (Å2)				
All atoms	20.55	48.67	53.6	43.0
Macromolecules	18.86	48.52	58.87	40.0
Solvent atoms	29.63	51.63	47.36	54.4
Other atoms	19.94		47.02	71.4
PDB ID	8PU2	8PU3	8PU4	8PU1

Supplementary Table 2. Experimental molecular weight parameters of ATfaRel2, FaRel2 and their variants.

Samples	SEC Molecular Weight (kDa)	Molecular Weight (kDa)	Oligomeric state
ATfaRel2	11.7	9.5	monomer
FaRel2^{Y128F}	23.5	24.8	monomer
FaRel2^{Y128F} : ATfaRel2	70.8	68.5	heterotetramer (2:2)
FaRel2^{Y128F} : ATfaRel2^{R47A}	51.3	59.1	heterotrimer (2:1)
ATfaRel2^{R47A}	10.8	9.4	monomer
ATfaRel2^{F59A}	8.0	9.4	monomer

Supplementary Table 3. Binding parameters determined by Isothermal Titration Calorimetry (ITC). Experimentally determined binding thermodynamic parameters resulting from ITC measurements. (*) The two numbers separated by a slash were obtained from the two-site binding model and correspond to the two independent binding sites of FaRel2^{Y128F} recognised by ATfaRel2^{R47A}. N.D. stands for 'not detectable'. All of the presented titrations are background-subtracted.

Titrations	K_D (nM)	ΔH (kcal/mol)	$-T\Delta S$ (kcal/mol)	ΔG (kcal/mol)	Molar Ratio (n)
ATfaRel2 into FaRel2^{Y128F}	35.2	-5.4	-4.6	-10.0	1.1
ATfaRel2 into FaRel2$^{Y128F / \Delta 166\text{-}206}$	3250.0	3.3	-10.7	-7.4	1.2
ATfaRel2^{V43A} into FaRel2^{Y128F}	78.8	-7.1	-2.6	-9.7	0.97
ATfaRel2^{I45A} into FaRel2^{Y128F}	868	-5.6	-2.7	-8.3	0.91
(*) ATfaRel2^{R47A} into FaRel2^{Y128F}	33/750	-5.8/-3.4	-4.4/-5.0	-10.2/-8.9	0.43 / 0.75
ATfaRel2^{A50M} into FaRel2^{Y128F}	38	-4.3	-5.8	-10.1	1.1
ATfaRel2^{Y51A} into FaRel2^{Y128F}	1521	-6.0	-1.9	-7.9	0.9
ATfaRel2^{Y54A} into FaRel2^{Y128F}	8751	-10.0	3.1	-6.9	0.92
ATfaRel2^{F59A} into FaRel2^{Y128F}	2151	-20.6	12.9	-7.7	0.99
mRNA into *S. aureus* RelQ	922	20.6	-28.8	-8.2	0.94
E. coli tRNA^{Val} into RelQ	N.D.	N.D.	N.D.	N.D.	N.D.
tRNA^{Val} into FaRel2^{Y128F}	483	1.1	-9.7	-8.6	0.9
tRNA^{Val} into phage Phrann PhRel^{Y143F} (Gp29^{Y143F})	825	3.3	-11.3	-8.0	1.1
tRNA^{Val} into *C. marina* FaRel^{Y175F}	6827	3.3	-10.4	-7.1	0.93
tRNA^{Val} into ATfaRel2:FaRel2^{Y128F}	460	1.6	-10.4	-8.8	1.0
APCPP into FaRel2^{Y128F}	2076	-3.8	-3.9	-7.7	0.9
APCPP into ATfaRel2:FaRel2^{Y128F}	41300	-17.7	11.7	-6.0	0.94

Supplementary Table 4. Strains, plasmids and oligonucleotide primers used in this study.

**Strain**	**Name**	**Description**	**Reference/source**
E. coli DH5α	F^{-} *endA1 glnV44 thi-1 recA1 relA1 gyrA96 deoR nupG purB20* φ80d*lacZΔM15 Δ*(*lacZYA-argF*)U169, *hsdR17*($r_K^{-}m_K^{+}$), λ^{-}	*E. coli* strain used for cloning	[37]
E. coli BW25113	F^{-}, *Δ*(*araD-araB*)*567*, *ΔlacZ4787*(*::rrnB-3*), λ^{-}, *rph-1*, *Δ*(*rhaD-rhaB*)*568*, *hsdR514*	wild-type *E. coli* BW25113	[38]
**Plasmid**	**Name**	**Description**	**Reference/source**
pBAD33	pBAD33	p15A, Cml^{R}, P_{BAD} promoter	[39]
pKK223-3	pKK223-3	ColE1, Amp^{R}, P_{Tac} promoter	[40]
pMG25	pMG25	pUC *lacIq* $P_{A1/04/03}$ promoter, Amp^{R}	41
VHp277	pBAD33-*faRel2*	*Coprobacillus* sp. D7 FaRel2 toxin expressed under the control of P_{BAD} promoter	[5]
VHp278	pKK223-3-*aTfaRel2*	*Coprobacillus* sp. D7 ATfaRel2 expressed under the control of P_{Tac} promoter	[5]
VHp1199	pMG25-*aTfaRel2*	ATfaRel2 expressed under the control of $P_{A1/04/03}$ promoter	This work
VHp1200	pMG25-*aTfaRel2 V43A*	ATfaRel2 V43A expressed under the control of $P_{A1/04/03}$ promoter	This work
VHp1201	pMG25-*aTfaRel2 I45A*	ATfaRel2 I45A expressed under the control of $P_{A1/04/03}$ promoter	This work
VHp1202	pMG25-*aTfaRel2 A50M*	ATfaRel2 A50M expressed under the control of $P_{A1/04/03}$ promoter	This work
VHp1203	pMG25-*aTfaRel2 Y54A*	ATfaRel2 Y54A expressed under the control of $P_{A1/04/03}$ promoter	This work
VHp1225	pKK223-3-*aTfaRel2 V43A*	ATfaRel2 V43A expressed under the control of P_{Tac} promoter	This work

VHp1227	pKK223-3-*aTfaRel2 I45A*	ATfaRel2 I45A expressed under the control of P_{Tac} promoter	This work
VHp1228	pKK223-3-*aTfaRel2 A50M*	ATfaRel2 A50M expressed under the control of P_{Tac} promoter	This work
VHp1229	pKK223-3-*aTfaRel2 Y54A*	ATfaRel2 Y54A expressed under the control of P_{Tac} promoter	This work
VHp1236	pMG25-*aTfaRel2 Y51A*	ATfaRel2 Y51A expressed under the control of $P_{A1/04/03}$ promoter	This work
VHp1366	pMG25-*aTfaRel2 R47A*	ATfaRel2 R47A expressed under the control of $P_{A1/04/03}$ promoter	This work
VHp1367	pMG25-*aTfaRel2 Y54A*	ATfaRel2 Y54A expressed under the control of $P_{A1/04/03}$ promoter	This work
	pMG25-*aTfarel2 Y57A*	ATfaRel2 Y57A expressed under the control of $P_{A1/04/03}$ promoter	This work
VHp1368	pMG25-*aTfaRel2 F59A*	ATfaRel2 F59A expressed under the control of $P_{A1/04/03}$ promoter	This work
VHp364	pET His-TEV-*aTfaRel2*	N-terminally His_6-TEV-tagged ATfaRel2 antitoxin expressed under the control of T7 promoter	[5]
	pET-His1a-*aTfaRel2 V43A*	N-terminally His_6-TEV-tagged V43A ATfaRel2 antitoxin expressed under the control of T7 promoter	This work
	pET-His1a-*aTfaRel2 I45A*	N-terminally His_6-TEV-tagged I45A ATfaRel2 antitoxin expressed under the control of T7 promoter	This work
	pET-His1a-*aTfaRel2 A50M*	N-terminally His_6-TEV-tagged A50M ATfaRel2 antitoxin expressed under the control of T7 promoter	This work
	pET-His1a-*aTfaRel2 Y54A*	N-terminally His_6-TEV-tagged Y54A ATfaRel2 antitoxin expressed under the control of T7 promoter	This work
	pET-His1a-*aTfaRel2 Y51A*	N-terminally His_6-TEV-tagged Y51A ATfaRel2 antitoxin expressed under the control of T7 promoter	This work

	pET-His1a-*aTfaRel2 R47A*	N-terminally His_6-TEV-tagged R47A ATfaRel2 antitoxin expressed under the control of T7 promoter	This work
	pET-His1a-*aTfaRel2 F59A*	N-terminally His_6-TEV-tagged F59A ATfaRel2 antitoxin expressed under the control of T7 promoter	This work
VHp971	pBAD33- *paSpo*	PaSpo SAH from *Salmonella* phage SSU5 expressed under the control of P_{BAD} promoter	This work
	pET-28b *His10 Sumo-FaRel2 Y128F*	N-terminally His_{10}-SUMO-tagged Y128F FaRel2 toxin expressed under the control of T7 promoter	This work
	pET-28b *His10 Sumo-FaRel2 Y128F / Δ166-206*	N-terminally His_{10}-SUMO-tagged Y128F FaRel2 toxin, truncated at residue 166 to remove the C-terminal dimerization domain, expressed under the control of T7 promoter	This work
	pET-28b *His10 SUMO FaRel Y175A*	N-terminally His_{10}-SUMO-tagged Y175A FaRel toxin expressed under the control of T7 promoter	This work
VHp83	pET-21a *RelQ (E. faecalis*)	C-terminally His_6-tagged SAS *E. faecalis* RelQ expressed under T7 promoter	[42]
